# Supplementary material for: Surgical Management of Hemorrhoidal Disease in Inflammatory Bowel Disease: A Systematic Review with Proportional Meta-Analysis
Source: J Clin Med. 2022 Jan 28;11(3):709. doi: 10.3390/jcm11030709 (PMC8837177; doi:10.3390/jcm11030709)

Supplementary Table S1. Search strategy

PubMed	(((inflammatory bowel disease[MeSH Terms])) OR (crohn disease[MeSH Terms])) OR (ulcerative colitis[MeSH Terms]) AND ((((((hemorrhoidectomy[MeSH Terms]) OR (hemorrhoidal artery ligation[MeSH Terms])) OR (hemorrhoidal stapler[MeSH Terms])) OR (hemorrhoidal dearterialization[MeSH Terms])) OR (sclerotherapy[MeSH Terms])) OR (rubber band ligation[MeSH Terms])) OR (infrared coagulation[MeSH Terms])<br><br>AND<br><br>((((((((postoperative complication[MeSH Terms]) OR (morbidity[MeSH Terms])) OR (pain, postoperative[MeSH Terms])) OR (mortality[MeSH Terms])) OR (quality of life[MeSH Terms])) OR (recurrence[MeSH Terms])) OR (hemorrhage, postoperative[MeSH Terms])) OR (wound infection, postoperative[MeSH Terms])) OR (anorectal stenosis[MeSH Terms])
Embase	('inflammatory bowel disease'/exp OR 'inflammatory bowel disease' OR 'inflammatory bowel diseases' OR 'crohn disease'/exp OR 'crohn disease' OR 'crohn`s disease' OR 'crohns disease' OR 'cleron disease' OR 'enteritis regionalis' OR 'intestinal tract, regional enteritis' OR 'morbus crohn' OR 'regional enteritis' OR 'regional enterocolitis' OR 'ulcerative colitis'/exp OR 'chronic ulcerative colitis' OR 'colitis ulcerativa' OR 'colitis ulcerosa' OR 'colitis ulcerosa chronica' OR 'colitis, mucosal' OR 'colitis, ulcerative' OR 'colitis, ulcerous' OR 'colon, chronic ulceration' OR 'histiocytic ulcerative colitis' OR 'mucosal colitis' OR 'ulcerative colitis' OR 'ulcerative colorectitis' OR 'ulcerative procto colitis' OR 'ulcerative proctocolitis' OR 'ulcerous colitis')<br><br>AND<br><br>('hemorrhoidectomy'/exp OR 'excision, hemorrhoid' OR 'haemorrhoidectomy' OR 'hemorrhoid excision' OR 'hemorrhoidectomy' OR 'hemorrhoidopexy' OR 'stapled hemorrhoidectomy' OR 'stapled hemorrhoidopexy' OR 'whitehead operation' OR 'hemorrhoidal artery ligation'/exp OR 'hemorrhoidal stapler'/exp OR 'haemorrhoidal stapler' OR 'haemorrhoidal surgical stapler, reusable' OR 'haemorrhoidal surgical stapler, single-use' OR 'hemorrhoidal stapler' OR 'hemorrhoidal surgical stapler' OR 'hemorrhoidal surgical stapler, reusable' OR 'rectal vessel stapler' OR 'reusable haemorrhoidal surgical stapler' OR 'reusable hemorrhoidal surgical stapler' OR 'single-use haemorrhoidal surgical stapler' OR 'transanal hemorrhoidal dearterialization'/exp OR 'sclerotherapy'/exp OR 'sclerosation' OR 'sclerosing injection therapy' OR 'sclerosing therapy' OR 'sclerotherapy' OR 'sclerozation' OR 'rubber band ligation'/exp OR 'infrared coagulation'/exp) AND ('complication'/exp OR 'complication' OR 'complications' OR 'morbidity'/exp OR 'disease frequency' OR 'disease incidence' OR 'disorder incidence' OR 'morbidity' OR 'morbidity pattern' OR 'morbidity rate' OR 'morbidity risk' OR 'rate, morbidity' OR 'mortality'/exp OR 'excess mortality' OR 'mortality' OR 'mortality model' OR 'quality of life'/exp OR 'hrql' OR 'health related quality of life' OR 'life quality' OR 'quality of life' OR 'pain'/exp OR 'acute pain' OR 'deep pain' OR 'lightning pain' OR 'nocturnal pain' OR 'pain' OR 'pain response' OR 'pain syndrome' OR 'treatment related pain' OR 'rescue therapy'/exp OR 'recurrent disease'/exp OR 'disease recurrence' OR 'periodic

	disease' OR 'recurrence' OR 'recurrent disease' OR 'relapsing disease' OR 'symptom flare up' OR 'incontinence'/exp OR 'elimination disorders' OR 'incontinence' OR 'incontinency' OR 'orthostatic incontinence' OR 'anal stenosis'/exp OR 'anal stricture'/exp OR 'bleeding'/exp OR 'abnormal bleeding' OR 'bleeding' OR 'bleeding complication' OR 'blood effusion' OR 'blood loss' OR 'capillary bleeding' OR 'haemorrhage' OR 'haemorrhage model' OR 'haemorrhagic activity' OR 'hemorrhage' OR 'hemorrhage model' OR 'hemorrhagia' OR 'hemorrhagic activity' OR 'spontaneous haemorrhage' OR 'spontaneous hemorrhage' OR 'infection'/exp OR 'accidental infection' OR 'acute infection' OR 'autoinfection' OR 'bacterial infections and mycoses' OR 'bacteroid infection' OR 'chain of infection' OR 'chronic infection' OR 'dormant infection' OR 'focal infection' OR 'inapparent infection' OR 'infection' OR 'infection mechanism' OR 'infection route' OR 'infection, focal' OR 'infections' OR 'infectious disease' OR 'infectivity' OR 'latent infection' OR 'route of infection' OR 'silent infection' OR 'simultaneous infection')
Scopus	(((inflammatory AND bowel AND disease)) OR (crohn AND disease)) OR (ulcerative AND colitis)<br><br>AND<br><br>((((((hemorrhoidectomy) OR (hemorrhoidal AND artery AND ligation)) OR (hemorrhoidal AND stapler)) OR (dearterialization)) OR (sclerotherapy)) OR (rubber AND band AND ligation)) OR (infrared AND coagulation) AND ((((((((complication) OR (morbidity)) OR (pain)) OR (mortality)) OR (quality AND of AND life)) OR (recurrence)) OR (bleeding)) OR (infection)) OR (anal AND stenosis)

Supplementary Figure S1. Proportional meta-analysis of No. patients with Crohn's disease / Total Patients.

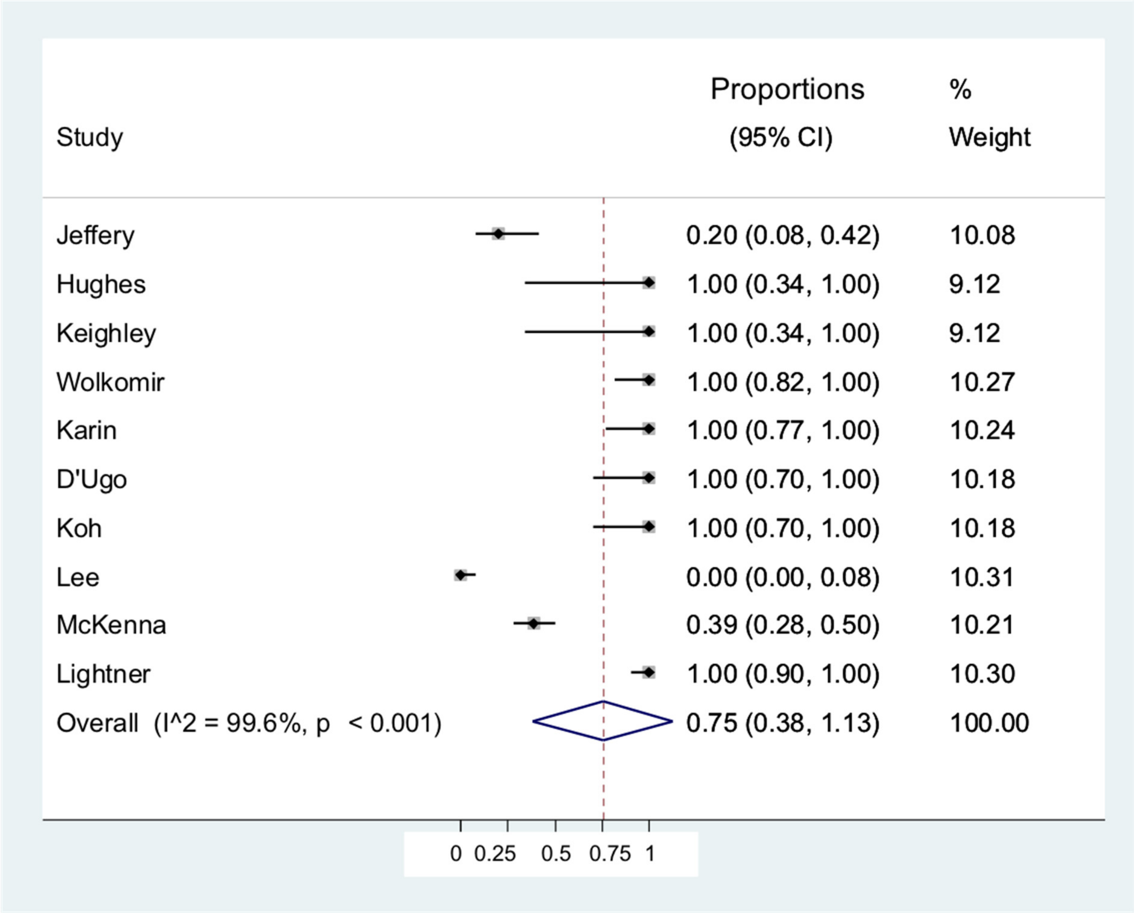

Supplementary Figure S2. Studies included in the proportional meta-analysis of complications (No. complicated / Total Patients)

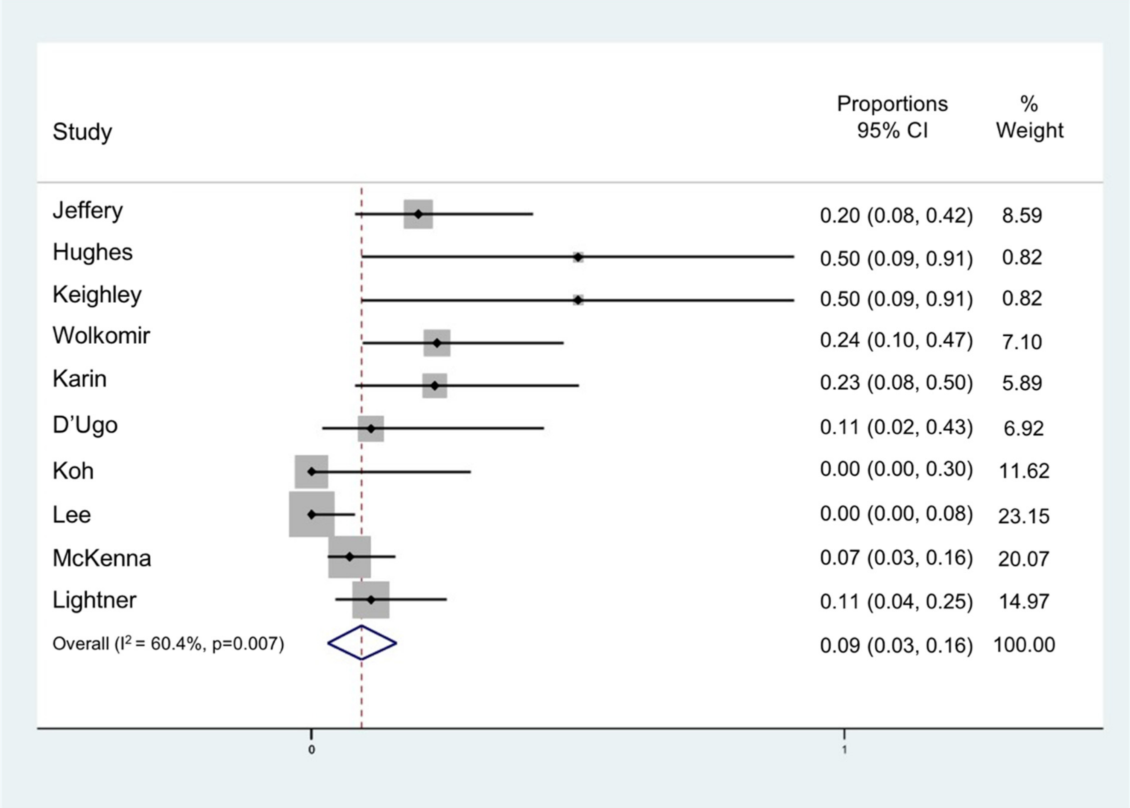

Supplementary Figure S3. Johanna Briggs Institute Critical Appraisal Checklist for Case Reports and Case Series

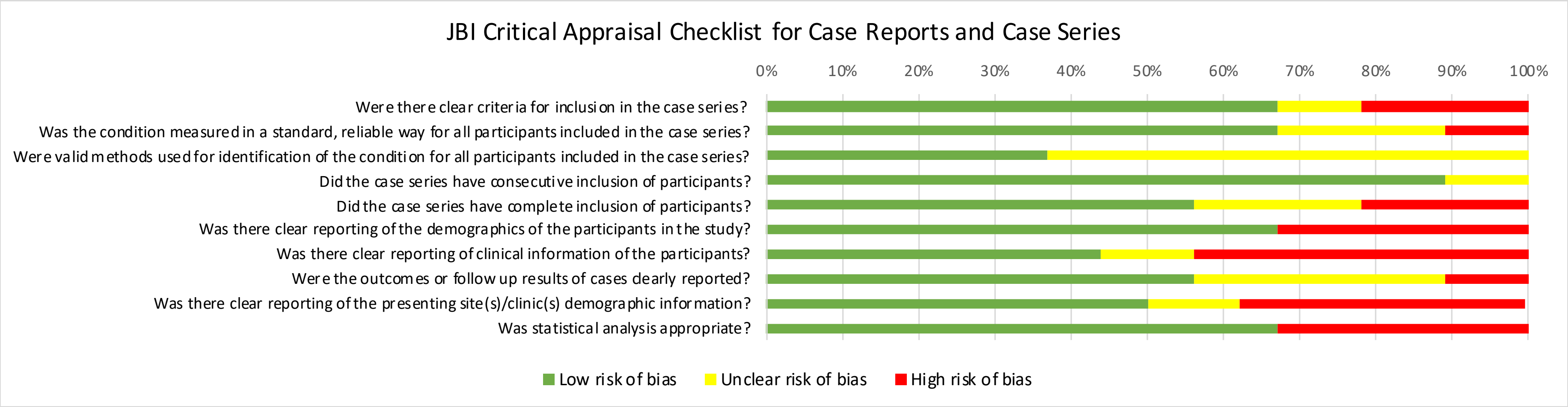

Supplement: Supplementary file 1 [file jcm-11-00709-s001.zip › jcm-1550891-supplementary.pdf]
